# QTL Mapping of Sex Determination Loci Supports an Ancient Pathway in Ants and Honey Bees

**DOI:** 10.1371/journal.pgen.1005656

**Published:** 2015-11-06

**Authors:** Misato O. Miyakawa, Alexander S. Mikheyev

**Affiliations:** 1 Ecology and Evolution Unit, Okinawa Institute of Science and Technology Graduate University, Tancha, Onna-son, Japan; 2 Research School of Biology, Australian National University, Canberra, Australian Capital Territory, Australia; New York University, UNITED STATES

## Abstract

Sex determination mechanisms play a central role in life-history characteristics, affecting mating systems, sex ratios, inbreeding tolerance, *etc*. Downstream components of sex determination pathways are highly conserved, but upstream components evolve rapidly. Evolutionary dynamics of sex determination remain poorly understood, particularly because mechanisms appear so diverse. Here we investigate the origins and evolution of complementary sex determination (CSD) in ants and bees. The honey bee has a well-characterized CSD locus, containing tandemly arranged homologs of the transformer gene [complementary sex determiner (*csd*) and feminizer (*fem*)]. Such tandem paralogs appear frequently in aculeate hymenopteran genomes. However, only comparative genomic, but not functional, data support a broader role for *csd*/*fem* in sex determination, and whether species other than the honey bee use this pathway remains controversial. Here we used a backcross to test whether *csd*/*fem* acts as a CSD locus in an ant (*Vollenhovia emeryi*). After sequencing and assembling the genome, we computed a linkage map, and conducted a quantitative trait locus (QTL) analysis of diploid male production using 68 diploid males and 171 workers. We found two QTLs on separate linkage groups (*CsdQTL1* and *CsdQTL2*) that jointly explained 98.0% of the phenotypic variance. *CsdQTL1* included two tandem transformer homologs. These data support the prediction that the same CSD mechanism has indeed been conserved for over 100 million years. *CsdQTL2* had no similarity to *CsdQTL1* and included a 236-kb region with no obvious CSD gene candidates, making it impossible to conclusively characterize it using our data. The sequence of this locus was conserved in at least one other ant genome that diverged >75 million years ago. By applying QTL analysis to ants for the first time, we support the hypothesis that elements of hymenopteran CSD are ancient, but also show that more remains to be learned about the diversity of CSD mechanisms.

## Author Summary

Whether a developing embryo becomes male or female has significant downstream consequences. Depending on the species, sex can be determined by a wide variety of mechanisms. Sex determination systems can evolve rapidly, but how this occurs, and even how widespread the same mechanism is within a given taxonomic group, remains largely unknown. By experimentally mapping the sex determination architecture in the ant, *Vollenhovia emeryi*, we found that the well-characterized honey bee sex determination locus originated more than 100 million years ago. However, we also found an additional locus that has no homology to the first. Currently uncharacterized, this locus suggests that different species may use a variety of complementary sex determination mechanisms. Yet, core elements of the complementary sex determination machinery appear to be ancient.

## Introduction

One of the major developmental events faced by most organisms is whether they become male or female [1,2]. Mechanisms underlying this choice have major implications for a variety of life history traits, and even the risk of extinction. For example, chromosomal sex determination systems, such as XY or ZW, dictate 50:50 sex ratios in the offspring. Other systems, such as haplodiploidy or environmental sex determination, can in principle allow females complete control of the sex ratio. Some sex determination mechanisms even impose ecological costs under certain circumstances. For instance, under complementary sex determination (CSD), individuals heterozygous at one or more CSD loci become females, while those homo- or hemizygous become males [3] (*e*.*g*., aculeate Hymenoptera, such as ants, bees, and social wasps). Single-locus CSD systems carry a high penalty for inbreeding (up to 50%), since diploid males resulting from homozygosity at these loci are typically sterile [3], and under CSD, small populations can fall into an extinction vortex [4]. Sex determination systems can evolve rapidly in response to biotic and abiotic conditions that alter sex ratios, such as changes in temperature for environmental sex determiners [5], or in response to bacterial feminizing factors [6]. Consequently, one expects a well-modulated interplay between molecular mechanisms of sex determination, and the ecology of a particular species.

While molecular pathways underlying sex determination are diverse, they nonetheless contain highly conserved elements, but their evolutionary dynamics remain poorly understood. In particular, few examples of turnovers in sex determination mechanisms have been rigorously studied [7]. More specifically in insects, although a wide variety of sex determination mechanisms exist, many of them revolve around a core conserved pathway [1,8]. In the fruit fly (*Drosophila melanogaster*), where sex determination is best understood, this pathway involves the transformer (*tra*) gene, which regulates doublesex (*dsx*), a gene that initiates the female developmental pathway [9]. Interestingly, there is evidence that *tra* plays a role in sex determination in other species as well [1]. For example, in the jewel wasp, *Nasonia vitripennis*, sex determination relies on genomic imprinting, with *tra* mRNA being provided maternally [10,11]. By contrast, in honey bees (*Apis mellifera*), a homolog of *tra* called feminizer (*fem*) underwent duplication to give rise to the complementary sex determiner (*csd*) gene [12]. The two genes are located adjacent to each other, and *csd* acts upstream of *fem*, which in turn acts on *dsx*. However, with the exception of these two species, little is known about the mechanisms of sex determination and their evolution in other hymenopteran insects.

Although *tra* and *dsx* are widely distributed phylogenetically among insects [1], most of the evidence implying their importance has been indirect. Recent comparative genomics studies have noted that many hymenopteran species have tandem copies of *tra* (as does the honey bee) that show signatures of balancing selection and synteny conservation [13]. Because the tandem *tra* homologs are widespread, *csd/fem* was even proposed as the ancestral hymenopteran sex determination system [14]. Although the idea that there exists an ancient CSD mechanism is intriguing, thus far no functional data support this hypothesis. Rather, preliminary data from the fire ant (*Solenopsis invicta*) suggest that *tra/fem* do not play a role in CSD in this species [15]. Furthermore, even more recent comparative genomics work has disputed the hypothesis that *tra*/*fem* are generally involved in aculate hymenopteran CSD, suggesting separate origins of CSD pathways, and pointing out that in the absence of functional studies, comparative data have limitations [16,17]. Here we test the whether *tra*/*fem* are involved in sex determination in hymenopteran insects other than the honey bee by mapping CSD loci in the ant *Vollenhovia emeryi*.

**Fig 1:**
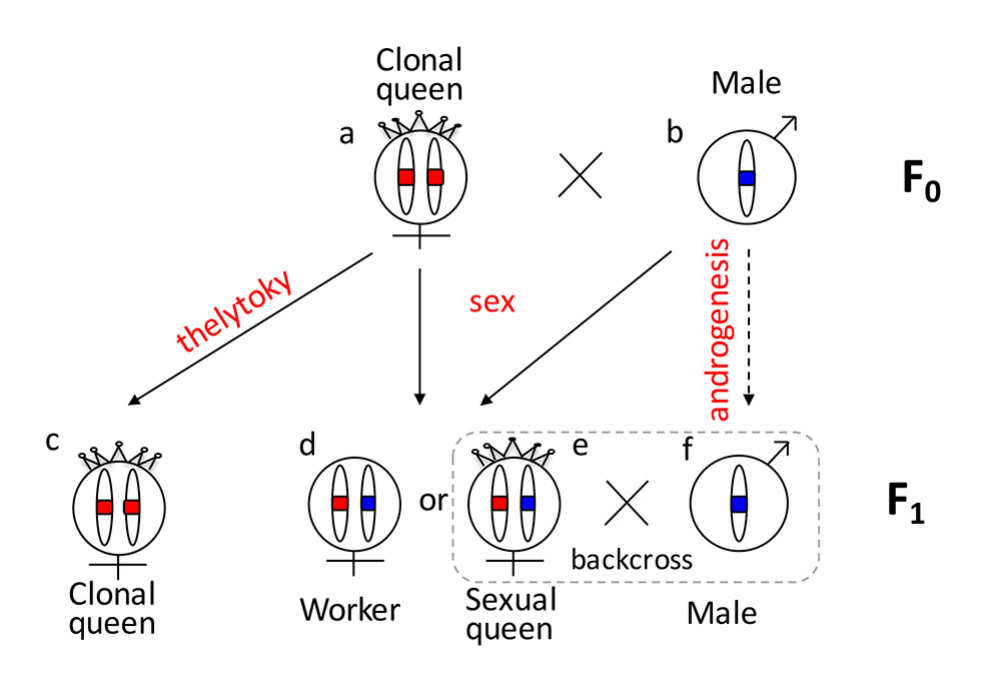
Graphical summary of the unusual reproduction system of *V*. *emeryi* and the experimental design. Females and males are diploid and haploid, respectively, as indicated by the number of oval cartoon chromosomes, which are colored red and blue in queens (a) and males (b) in the parental generation (F_0_). *V*. *emeryi* reproduce clonally (via thelytoky and androgenesis), as well as sexually, with all possible offspring shown in F_1_. These reproductive modes produce four types of offspring: clonal queens by thelytoky (c), queens and workers by sexual reproduction (d and e), and androgenetically produced clonal males (f) that are identical to their fathers despite developing from queen-laid eggs [22] [20,21]. The experimental cross (encircled by a dashed line), which is genetically identical to a backcross, mated sexually produced queens (e) and clonal males (f) in the F_1_ generation and scored diploid male production in the F_2_ generation. The F_2_ offspring are not depicted in this diagram.

One reason that functional evidence for the role of *tra*/*fem* has been hard to obtain is that many ants, bees, and social wasps have complex life cycles. As a result, it is impossible to rear multiple generations or to cross them experimentally for mapping studies. Species that can be crossed are often adapted to routine inbreeding, and do not produce diploid males [18,19], thus lacking phenotypic diversity necessary for mapping studies. *V*. *emeryi* is particularly suited to linkage mapping analysis because of its unusual reproductive system (Fig 1), which involves parthenogenetic reproduction by queens, and androgenetic reproduction by males, the latter arising from eggs that lack the queen’s genome [20,21]. In addition, workers and some queens are produced sexually [22]. Sexually produced queens can be crossed with the paternal clone in the genetic equivalent of a classic backcross, an experimental design we used to investigate the genetics of diploid male production in this species (Fig 1).

*V*. *emeryi* is one of the few ants that can be experimentally crossed in the lab and reared across several generations. It provides an excellent model system to address a range of biological questions, including mechanisms of sex determination, genetic caste determination, and social parasitism [21,23,24]. Here we present a wide range of genomic resources for this species, including a genome, transcriptome, linkage map, and population genomic data, and use them to investigate the mechanism of sex determination. Using QTL mapping we show that worker sex determination in *V*. *emeryi* relies on multi-locus CSD (ml-CSD) with two loci, one of them at the same position as duplicated *tra* homologs, supporting the ancient role of this sex determination mechanism. However, we also uncovered another locus, which is unrelated to the first, and which illustrates the diversity of hymenopteran CSD systems.

## Results

### Genome assembly and annotation

The genome assembly totaled 287,900,827 bp, including 19,131,583 gaps (47.0X coverage). It had 13,258 scaffolds (N50 1,346,088) and 23,916 contigs (N50 32,417). As evaluated with BUSCO, the genome assembly was largely complete, with only 16 out of 2,675 (0.59%) universal, single-copy orthologs missing, and 64 (2.4%) partial genes. The genome annotation contained 14,870 coding genes, of which 9,239 had a known protein with an alignment covering 50% or more of the query, and 2,671 had an alignment covering 95%. The complete annotation report is available from NCBI (*V*. *emeryi* Annotation Release 100). This genome was a major improvement over the previous genome version, which was based only on shotgun Roche 454 data [24], and could be used for linkage mapping.

### Diploid male production from inbred and outbred crosses

A quarter (27.1 ± 8.91% SD) of all offspring in inbred crosses were diploid males, while the rest were workers, and a single queen (Table 1). By contrast, of the 1,742 offspring produced by 17 queens mated to males from other populations (outbred crosses) there were no diploid males after ten months of laboratory rearing (S1 Table). The ratio of diploid males produced in inbred crosses was not significantly different from 25%, as predicted for a model with two independent sex determination loci.

**Table 1:** Offspring produced by sib-mated queens.

	Number of offspring				
Queen ID	Queens	Workers	Diploid males	Total	Diploid males (%) ± 95% C.I.
1	0	91	29	120	24.2 ± 7.7
2	0	102	37	139	26.6 ± 7.3
3	0	84	30	114	26.3 ± 8.1
4	0	45	12	57	21.1 ± 10.6
5	1	51	13	65	20.0 ± 9.7
6*	0	20	16	36	44.4 ± 16.2

A quarter of the offspring were diploid males, which do not produce functional sperm (S1 Fig). By contrast, no diploid males were produced by 17 queens mated to males from other populations. Confidence intervals were computed using a one-sample proportion test. Except for one colony, where the queen died prematurely, all others produced diploid male ratios not significantly different from the 25%, which is expected under a two-locus sex determination model, but different from the 50% expected under a single-locus model.

* The queen died about one month into the experiment

### Sterility of diploid males

While seminal vesicles of androgenetic haploid males contained sperm, those of diploid males did not, even five months after eclosion, indicating that diploid males were sterile (S1 Fig), and suggesting that inbred queens must invest a quarter of their resources producing reproductively useless males. Correspondingly, inbred queens also produced about 25% fewer workers. Despite the fact that diploid males were immediately removed from the colonies during the experiment, the number of emerged workers per month produced by inbred queens was significantly lower than for outbred queens (8.3 ± 3.06 *vs*. 13.9 ± 4.80, P < 0.05, F_1,21_ = 6.81, one-way ANOVA). This suggests that colonies do not compensate for production of diploid males in other ways, such as increasing worker production.

### Linkage map and QTL analysis

**Fig 2:**
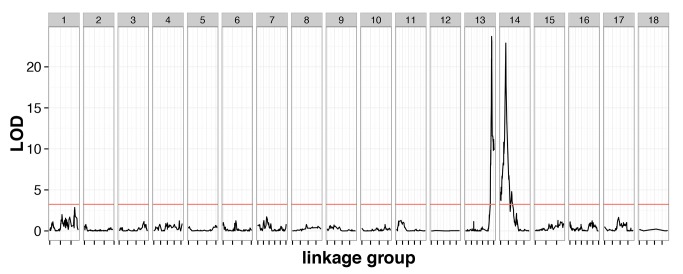
Linkage mapping confirms the existence of two-locus CSD in *V*. *emeryi*. Marker locations are shown by short dashes along the x-axis of the plot. A red line shows the permuted 95% significance level of the LOD score. This genome scan found two loci, named *CsdQTL*1 and *CsdQTL*2, on LG13 and LG14, respectively. These two loci and their interaction explained 98.0% of the observed phenotypic variance. The two-locus model obtained by QTL analysis is consistent with sex ratio data from the experimental crosses, which find a quarter of diploid males in the F_2_ offspring (Table 1).

Note: The automated NCBI annotation fused the two *tra* homologs at *CsdQTL1*. Unfortunately, this error will remain in the genome viewer for the foreseeable future (http://goo.gl/oNp5Jq). However, the NCBI RefSeq database has been updated with the manually corrected nucleotide sequences (*traA*: NM_001310035.1 and *traB*: NM_001310036.1).

The cross contained 239 individuals genotyped at 3,541 markers with 1.9% missing data, and a genotype error rate of 0.26%, inferred from quality scores (S1 Data). These markers clustered into 18 linkage groups, consistent with previous karyotype estimates [25]. A genome scan identified two QTLs with large phenotypic effect (*CsdQTL1* and *CsdQTL2*), located on linkage groups 13 and 14, which jointly accounted for almost all of the phenotypic variance in diploid male production (98.0%) (Fig 2). The two regions can be explored interactively using the NCBI genome browser (*CsdQTL1*: http://goo.gl/oNp5Jq, *CsdQTL2*: http://goo.gl/8x3MLe). Located on LG14, *CsdQTL1* includes three tightly linked [0 centimorgans (cM) apart] markers spanning 38kb and located 20kb from two tandem *tra* homologs. This arrangement is typical of other ants [13,14]. *CsdQTL2* spans 3.2 cM and covers a range of at least 236kb on scaffold NW_011967112.1, which is spanned by eight markers, co-located at two positions on the linkage map. It also includes one marker from scaffold NW_011967235.1, which probably resulted from a scaffolding error, since the rest of that scaffold is on LG2. Although QTL analysis does not explicitly consider the effect of heterozygosity on a phenotype, diploid male phenotypes were associated with homozygosity at these loci (S1 Data), consistent with the proposed mechanism of sex determination. There was no homology between *CsdQTL1* and *CsdQTL2*, suggesting that these loci did not arise by duplication.

### Population genomic investigation of CSD QTLs

**Fig 3:**
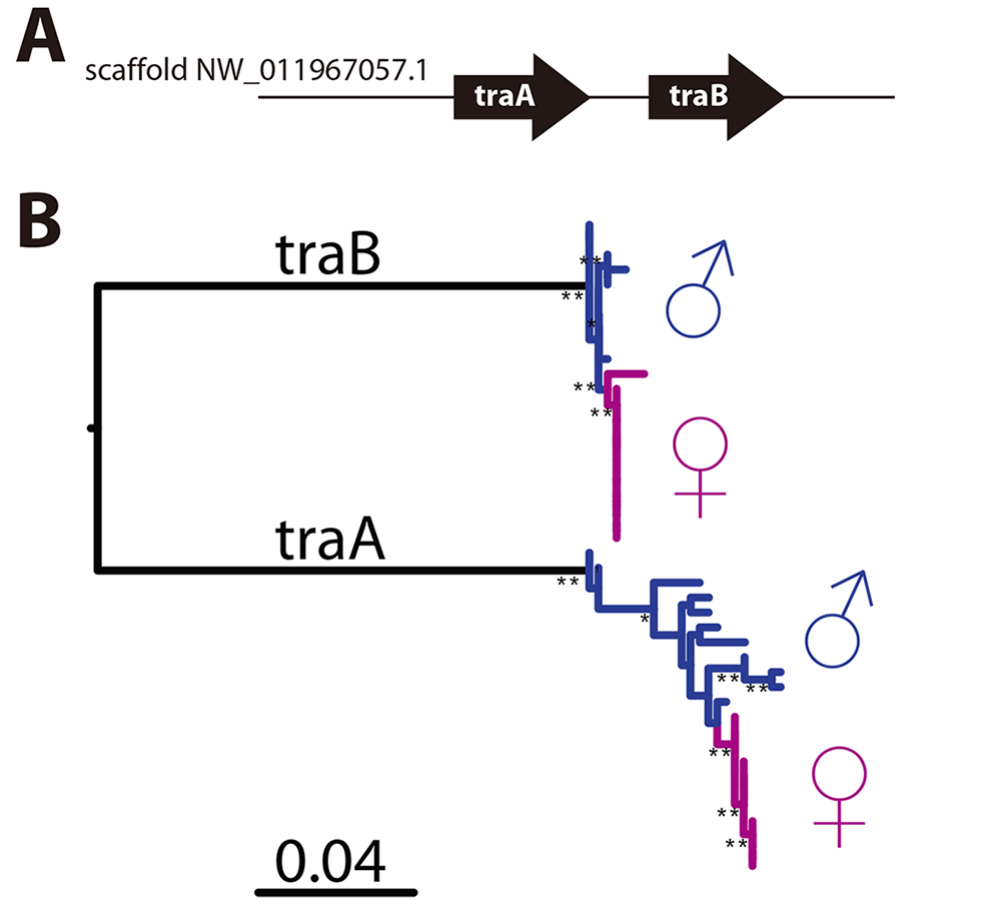
*CsdQTL1* contains csd/*fem* with separate alleles in males and queen clones. (A) Locus *CsdQTL1* contained two tandem copies of *tra* homologs on the same scaffold. (B) Males and queens have different copies of both *traA* and *traB*, but the former gene is more diverse. Nodes with 100% bootstrap support are shown with double asterisks and nodes with 90–99% support with single astersks. These data suggest that the *traA* and and *traB* copies diverged before male and female clones separated from each other. As a result, each mating produces workers heterozygous at this locus, which was also consistent with linkage mapping and diploid male production data. The *traA* gene has higher diversity than *traB* and weak evidence of diversifying selection (S3 Fig), suggesting by analogy with honey bees, that this is the homolog of *csd*, while *traB* is the homolog of *fem*. Scale bar is in substitutions per site.

*CsdQTL1* contains two *tra* homologs (*traA* and *traB*, NCBI NM_001310035.1 and NM_001310036.1, Fig 3A). Re-sequencing of this QTL region in 10 males and 11 queens showed that the sexual clones differ in protein sequence at both *traA* and *traB* (Fig 3B). Divergence of these genes preceded the separation of male and female clones (Fig 3, S2 Fig). In general, the two *tra* homologs are more similar to each other within a species, relative to other species, making it difficult to determine putative *csd* and *fem* genes [13,14], a pattern that also holds true in *V*. *emeryi* (S2 Fig). Interestingly *traA* has much higher diversity than does *traB*. In honey bees, *fem* and *csd* experience stabilizing and diversifying selection, respectively, and copies of *csd* are more numerous [12,26]. Although this effect is much less pronounced in *V*. *emeryi*, MEME analysis found two sites under episodic diversifying selection in *traA*, but found no such site in *traB* (S3 Fig). By analogy with honey bees, it seems that given its higher peptidyl variability, and some evidence of diversifying selection, *traA* may be the homolog of *csd*. However, queens have no non-synonymous heterozygosity in either *traA* or *traB*, and therefore heterozygosity at this locus is not required for female determination in queens. Unlike *CsdQTL1*, we found no obvious candidate CSD genes for *CsdQTL2*, which spans 15 annotated genes (Table 2). None of the genes found in this region are homologous to *CsdQTL1*, indicating that ml-CSD in *V*. *emeryi* did not evolve by duplication. A NCBI BLASTN search using default parameters found that the entire scaffold containing *CsdQTL2* was highly conserved (79% sequence identity and perfect synteny) in the genome of the little fire ant (*Wasmannia auropunctata*) (NCBI accession NW_012026774). There were also hits to fire ant scaffolds, but they are not contiguous enough to draw definite conclusions about structural conservation.

**Table 2:** Genes located at *CsdQTL2*.

NCBI Gene ID	Description
LOC105559778	KN motif and ankyrin repeat domain-containing protein
LOC105559779	BET1 homolog
LOC105559781	probable ribosome production factor 1
LOC105559780	THUMP domain-containing protein 3-like
LOC105559782	cysteine-rich with EGF-like domain protein 2
LOC105559784	coatomer subunit alpha
LOC105559783	uncharacterized
LOC105559785	host cell factor
LOC105559786	probable myosin heavy chain ECU04_1000
LOC105559787	GTP-binding protein Rit2
LOC105559788	ero1-like protein
LOC105559790	protein dispatched-like
LOC105559789	protein dispatched-like
LOC105559791	Bardet-Biedl syndrome 1 protein
LOC105559793	lachesin-like

None of the genes at *CsdQTL2* are homologs of *tra*, suggesting that the molecular machinery of CSD is different at this locus. None of the genes at this locus are similar to other known sex determination genes.

### Population genetic analysis using microsatellite markers

Sequences of microsatellite markers developed from RAD-tag data, population-level summary statistics, and raw data can be found in S2 Data and S1–S4 Tables. *V*. *emeryi* in its native range (in Japan) exists in isolated populations with preponderantly one male and one queen clone in each one (S4 Fig, S4 Table). Even though isolated patches where these ants are found were sometimes separated by only a few meters, each one had a different pair of clones, indicating minimal gene flow between them (S4 Fig, S3 Table). By contrast, male and female clones were the same in experimental invasive US populations, separated by about 10 km, suggesting regionally low genetic diversity (S4 Fig, S3 Table).

## Discussion

**Fig 4:**
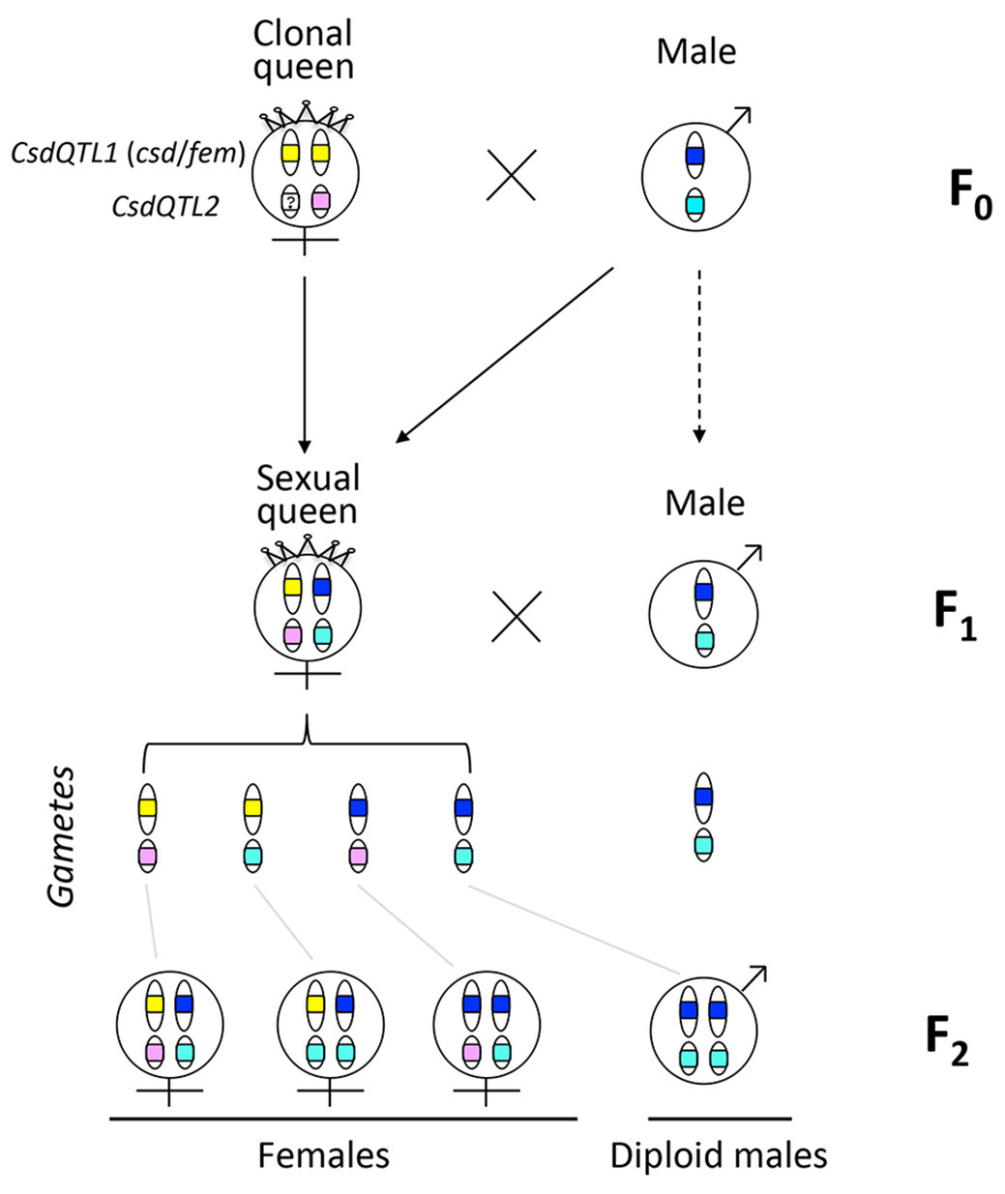
Graphical summary of the results, with different CSD alleles color-coded. QTL mapping (Fig 2) and F_2_ diploid male ratios (Table 1) both support a two-locus model of sex determiation in the workers. *CsdQTL1*, which has homologs of *csd* and *fem*, has diverged between male and female clones (Fig 3), assuring heterozygosity at that locus in F_1_ females. Both *tra* homologs are homozygous in clonal queens (F_0_), suggesting that if queens use the same sex determination mechanism as do workers, *CsdQTL2* should be heterozygous. However, none of the exonic regions at *CsdQTL2* are heterozygous in all of the queen samples. If this result is not a technical artifact, it suggests either thtat queens use a separate sex detemination mechanism, or that the sex determination mechanism in *V*. *emeryi* does not depend on heterozygosity in protein sequence at *CsdQTL2*. In any case, the molecular mechanism at *CsdQTL2* remains a mystery.

Sex ratio data and QTL mapping analysis both show that *V*. *emeryi* has two unlinked sex determination loci (*CsdQTL1* and *CsdQTL2*) (Figs 3 and 4, Table 1). The *CsdQTL1* locus has duplicated *tra* homologs, as is typical of ants [13] (S2 Fig). This configuration resembles the sex determination locus of honey bees, where *csd* is located adjacent to *fem*, with both genes acting in the sex determination pathway [12,27]. As ants and bees diverged more than 100 million years ago [28], sex determination in honey bees and *V*. *emeryi* is probably homologous and has been conserved for at least this long. These data are consistent with a hypothesis based on comparative genomic data, which proposed that *csd*/fem form the core of an ancient pathway in Aculeata, or possibly even a more ancient hymenopteran [14], though investigations of other hymenopteran lineages will be necessary to confirm this hypothesis. It is possible that *csd*/*fem* evolves rapidly in species-specific ways as a result of frequent gene conversion [29]. Alternatively, CSD could have evolved separately in both lineages by convergent co-option and duplication of *fem* for CSD [16]. The latter scenario seems less likely, given the frequent co-occurrence of syntenic *tra* homologs across a range of hymenoptera [13,14] (S2 Fig), and would require a remarkable convergence in the evolutionary patterns and function of CSD loci in aculeate Hymenoptera.

### Population genomic analysis of sex determination loci

Population genomic analysis revealed that *V*. *emeryi* males and queens have different protein sequences at *CsdQTL1*, which ensures that workers are always heterozygous at this locus (Figs 3 and 4). One of the genes, *traA*, had much higher protein diversity and weak evidence of diversifying selection, suggesting that it may be the *csd* homolog. Still, levels of allelic diversity in *traA* and selection were much lower than in the honey bee. This may be the result of *V*. *emeryi*’s unusual clonal reproductive system, where males and queens form separate lineages, and clonality fixes diversity at *CsdQTL1* between them (Figs 1 and 4). Since every mating is thus guaranteed to produce a heterozygous combination of CSD loci in the F_1_ offspring, we would not expect diversifying selection to act upon them strongly. Interestingly, the F_0_ queens did not show protein-level differences at either *traA* or *traB*. This suggests either a different sex determination mechanism in the clonal queens *vs*. their sexually produced daughters, or that the clonal queens are fixed for heterozygosity at some locus linked to *CsdQTL2* (Fig 4).

### How does the second locus work?

When Crozier proposed the existence of ml-CSD [30], he envisioned that the different loci evolve by duplication and act together as a heteropolymers, similarly to hemoglobin, which consists of paralogous α and β subunits. This does not appear to be the case, as the QTL loci share no homology and appear to act independently (Table 1, Fig 2). However, the actual genomic locus where *CsdQTL2* is located appears quite old, being shared with *W*. *auropunctata*, representing more than 75 million years of evolutionary divergence [31]. It is presently unclear whether *W*. *auropunctata* or other ants use this locus for sex determination, but the fact that it is conserved offers an opportunity for future comparative analysis. QTL data alone cannot provide further functional insight into the actual molecular mechanisms encoded by *CsdQTL2*. Future work focusing on sex-specific splicing of genes at this locus in F_2_ workers *vs*. diploid males during development may shed additional light on the mechanism.

### Clonality: A strategy to overcome the limitations of CSD?

Microsatellite analysis shows that in the native range, patches of *V*. *emeryi* are highly genetically differentiated (on the scale of meters), with each patch being dominated by different pairs of male and female clones (S4 Fig, S3 Table, S4 Table). Though this should create high potential for inbreeding, a decade of genetic studies has not detected diploid males [21,22,32,33]. The absence of diploid males is not a result of their effective elimination by the colonies, since they are readily produced in our experimental crosses, suggesting that clonality in *V*. *emeryi*, and perhaps in other ants with the same reproductive strategy may effectively prevent inbreeding [34]. Completely clonal ants, which often have low rates of recombination, may also retain heterozygosity at sex determination loci [35,36]. Thus, clonality may represent an evolutionary strategy to circumvent limitations posed by ancestral CSD. Many clonal ants are also invasive or live in human-associated habitats [37], suggesting that clonality may also facilitate anthropogenic spread. For instance, most invasions by the highly invasive little fire ant (*W*. *auropunctata*) are initiated by single queen clone introductions [38], which may also be the case for the introduction of *V*. *emeryi* into the United States. Finally, CSD may explain why queens in species such as *V*. *emeryi* and *W*. *auropunctata* mate to clonal male lineages rather than producing sons. Separate male and queen clones maintain fixed heterozygosity at CSD loci in workers, allowing them to be produced sexually but without the possibility of inbreeding (although see [39] for alternative view).

### Implications for the evolution of CSD loci

More recent work has challenged the original suggestion [14] that *csd* and *fem* form the core of an ancient hymenopteran CSD system [16,17]. Critics principally argue that *fem* and *csd* homologs do not experience consistent selective pressures across hymenopteran lineages; thus they are not likely part of a conserved CSD pathway. However, multi-locus CSD systems may reconcile the ancient function of *csd*/*fem* in CSD with lineage-specific differences. Since only one locus is necessary for sex determination, a ml-CSD system can collapse into a single-locus system if allelic diversity at the other loci is lost [40]. Thus, lineages can potentially converge on different single-locus CSD systems. This may have happened in the fire ant, which does not appear to use *tra*/*fem*, at least in the invasive range where it was studied [15], and where there was ample opportunity for allelic loss. Since ml-CSD is known from other Hymenoptera [40], and the *CsdQTL2* region is conserved, it is even possible that the mechanism encoded by *CsdQTL2* is part of ancestral CSD that was lost in the honey bee. Detailed mapping studies in other Hymenoptera including basal taxa will be needed to fully resolve the evolution of *csd*/*fem* and other CSD loci.

## Materials and Methods

### Samples used in the study

*V*. *emeryi* nests in slightly wet, fallen branches in secondary forests. Field surveys confirmed that all sites were composed of colonies containing clonal, short-winged queens [23]. Because queens do not have functional wings, colonies are typically propagated by budding after intra-colony mating, and occur in dense, but isolated patches, which we call ‘sites’ in the present study [41]. We collected live *V*. *emeryi* colonies from the native range at one site in Tokyo and four sites in Ishikawa Prefecture in Japan, from May to September 2013. We also genotyped invasive populations from three sites in near Rockville, Maryland, USA, collected in 2012. Experimental colonies were provided dry crickets, sugar water, and distilled water every other day. These colonies were kept in artificial plaster nests at 25 C, 50–60% humidity and a 12-hour light/dark cycle.

### Genome and transcriptome sequencing, assembly, and annotation

The reference genome was shotgun sequenced from two sets of PCR-free libraries (GS FLX+ library preparation kit for Roche, and TruSeq DNA PCR-free sample preparation kit for Illumina), and several mate-pair libraries (GS FLX paired-end kit (Roche)). One set of libraries, made from a single male clone, was sequenced on the Roche 454 FLX+ platform, yielding 6,216,952 reads containing 3.6 gigabases of sequence. Another set of libraries was prepared from another male using an Illumina TruSeq kit with 6 amplification cycles, and sequenced using an Illumina MiSeq in paired-end 2x300 mode. The two paired ends were subsequently merged using PEAR (parameters:—min-overlap 10 -n 200 -m 600 -p 0.0001) [42] to produce 23,078,792 (10.7 gigabases) high-quality super-reads (440 ± 80 bp), which are similar to 454 reads in length distribution. Both libraries were assembled using Newbler (v. 2.6), which was developed specifically for the 454’s medium-length reads (parameters: -large -m -cpu 10 -mi 95 -siom 390 -l 1000 -a 500 -urt -novs -a 1000) [43]. We also sequenced mate-paired libraries for scaffolding, which were prepared using Illumina kits (two 3.5 kb libraries, two 5.5 kb libraries, two 8.5 kb libraries and one 14 kb library). We sequenced approximately 600,000 read pairs in every library. The Newbler assembly was scaffolded using SSPACE (3.0) (parameters: -z 1000 -p 1 -x 1 -v 1) [44].

For purposes of annotation, we also sequenced RNA-seq libraries prepared from males, queens, and workers (5 replicates each) collected at sites A-D (S4 Fig). The libraries were prepared as in Aird *et al*. [45] and were sequenced on an Illumina HiSeq 2000 in paired-end 100 cycle mode. RNA-seq libraries yielded an average 7,086 ± 1,167 mb of data. Scaffolds were annotated using NCBI’s automated genome annotation pipeline, which takes advantage of species-specific RNA-seq data, as well as extensive protein homology data stored in GenBank [46].

### Experimental crosses

Colonies collected at site A (S4 Fig) were kept in the laboratory until new reproductives emerged. Wings of new reproductives were genotyped to classify them into two groups: short-winged queens produced through parthenogenesis, and long-winged queens produced sexually [22]. We crossed sexually produced queens, which had both parental genomes, with their brothers, which had only the paternal genetic contribution. This cross was equivalent to a classic backcross between inbred lines, and produced inbred F_2_ offspring (Fig 4).

For crosses, one to five queens were placed in small artificial nests with one male, and with a few workers for three days under constant light. After that, queens were isolated and kept in new artificial nests with 20 to 30 workers. Six of the twenty experimental queens mated with males and started to lay eggs. Eggs and larvae produced by sib-mated queens were transferred to other nursing colonies containing only workers, until offspring emerged. To compare rates of diploid male production for inbred and outbred colonies, we also set up 21 crosses between sexually produced queens from site A and males from three other sites (S4 Fig, Site B, C & D). Nineteen of the twenty-one experimental queens mated with males and started to lay eggs. Because two of the 19 mated queens died before laying eggs, 17 queens were used for experimental outbred crosses.

### Observation of testicular development and sperm production

To determine fertility of diploid males and haploid males, we monitored gonadal and accessory gland development from one to three months after eclosion, paying particular attention to sperm production. Six more diploid males were dissected five months after eclosion. In total, 32 lab-reared diploid and 16 haploid (lab-reared and field collected) males were randomly chosen and dissected.

Dissected testes and accessory glands were treated with 4% PFA for 30 min. After fixation, tissues were washed with 0.1% PBT (PBS and 0.1% Triton X-100) 5 times and mounted on VECTASHIELD Mounting Medium with DAPI (Vector Laboratories, Inc). Tissues under the cover glass were gently compressed during mounting and observed with a Zeiss Axio Scope LED at 400× magnification.

### Linkage and QTL mapping

RAD-tag libraries were prepared using the methodology in Tin *et al*. [47], including removal of duplicate reads. RAD-tag data were sequenced for 288 individuals on an Illumina HiSeq 2500 in single-end 50-bp mode. Raw data were sorted by barcode, and aligned to the *V*. *emeryi* genome assembly using bowtie2 [48]. Following alignment, duplicate reads were removed as in Tin *et al*. [47]. Genotype calls were made with FreeBayes using default parameters [49]. Raw genotypes were then filtered to include only bi-allelic sites with high quality (Q>40) using VCFTools [50]. For every locus we then sequentially dropped individuals with the most missing data, aiming to obtain the largest possible data set subject to the following constraints: (1) all genotype calls should have quality scores of at least 13 (95% accuracy), (2) no more than 5% missing data per locus, (3) minor allele frequency greater than 0.20. This data set was further filtered in R/QTL [51] to eliminate sites with segregation distortion (p<0.01) and four extremely homozygous individuals with (>20% homozygosity). Because our map was made from four families, we also checked to see whether there were families with non-segregating sites, which could skew allele frequencies; there were no such loci. The final data set contained 68 diploid males and 171 workers genotyped at 3,541 loci. The linkage map was computed using MSTMap (parameters: cut_off_p_value 0.00001, no_map_dist 15.0, no_map_size 2, missing_threshold .1, estimation_before_clustering yes, detect_bad_data yes, objective_function COUNT) [52]. QTL analysis was then carried out in R/QTL using a genome scan with a single QTL model and a binary response variable (see S1 Data and S1 Script).

### Population genomic analysis of *CsdQTL1*

In order to investigate species-level variation at this locus, we sequenced 10 male and 11 queen genomes from samples collected in Japan (eight sites including site A and C in S4 Fig), Korea (one site), and the United States (one site in S4 Fig). Libraries were prepared using Nextera XT kits, and sequenced on an Illumina HiSeq 2000 sequencer at an average mapped coverage of 25.5 ± S.D. 4.2. Variants were called using FreeBayes [49], and subjected to several levels of quality filtering. First we used VCFTools [50] to remove sites with quality scores less than 40, and with ≥30% missing data. We then filtered out sites containing indels and sites heterozygous in more than one male genome. We the used the FastaAlternateReferenceMaker module from GATK (v3.3) [53] to convert variant call files to sequence files for subsequent analysis. We then manually annotated *traA* and *tra*B gene models in Geneious (v. 8.1.3) [54] using its MAFFT (v. *7*.*017*) [55] plugin to create translation alignments of the genes. We then computed the best multiple likelihood protein tree with 100 bootstrap replicates using RAXML (v. 8.1.18) [56] (model PROTCATJTTF). Finally, we conducted MEME [57] analysis for episodic diversifying selection on *traA* and *traB* using the DataMonkey server, retaining default settings for the analysis [58].

### Development and amplification of microsatellite markers for population genetics

We screened for candidate microsatellite marker loci among perfect repeats showing polymorphism in our backcross RAD-tag data, producing 17 candidate loci. Native and invasive populations were genotyped using these loci, and those from other studies [59]. Because of variable polymorphism, not all loci were useful in all populations, but at least 11 microsatellite markers were used per population (S2 Table).

DNA extractions were performed using 5% Chelex solution, from bodies of queens without their gasters, from whole bodies of males, and from spermathecal contents of mated queens. Samples were incubated in 100 μL Chelex solution for 20 min at 95°C, and stored at 4°C. Instead of using sequence-specific fluorescent primers for each locus, we used universal M13 tails. PCR was performed in 10.05 μL volumes containing 0.2 μL of primer mix (2 μM forward primer with and M13(-21) tail at the 5´-end and 8 μM reverse primer), 1.2 μL universal fluorescent (FAM, HEX, or TAM) labeled M13 primer, 1 μL of diluted DNA, 1 μL of ExTaq 10x buffer, 1 μL of 2.5 mM dNTP, 0.05 μL of ExTaq enzyme, and 5.2 μL of RNAse-free water. PCR amplification conditions are as follows: 94°C (5 min), then 30 cycles at 94°C (30 s) / 58°C (45 s) / 72°C (45 s), followed by 8 cycles 94°C (30 s) / 53°C (45 s) / 72°C (45 s), and a final extension at 72°C for 10 min. Fluorescent PCR fragments were visualized by capillary electrophoresis on an ABI 3100xl Genetic Analyzer (Applied Biosystems). Genotypes were scored manually using GeneMarker [60].

## Supporting Information

S1 Fig Testes and accessory glands. (A) males produced by sib-mated queens and (B) androgenetic males, stained with DAPI (32 and 16 males of each kind were dissected). Only nuclei of glands were observed in diploid males, whereas sperm (fibrous tissue) could be seen in androgenetic haploid males. Diploid males perform no work and do not produce sperm, suggesting that they are a major cost to the colony. Scale bar represents 50μm.
(PNG)

S2 Fig Gene tree of *tra* homologs. In the honey bee the two transformer homologs are called *csd* and *fem*. In other species these genes are referred to as *tra* homologs, because they are not functionally characterized. The *tra* homologs of *V*. *emeryi* are more similar to each other than to those of other species, consistent with frequent gene conversion [13]. With the exception of *V*. *emeryi* genes, all other sequences are as in Fig 1 in [13]. The alignment was made using codon sequences in MAFFT [55] and the tree was computed using RAXML [56] under the GTR+G model with 100 bootstrap replicates. The scale bar is in substitutions per site.
(PDF)

S3 Fig MEME analysis of codons experiencing diversifying selection in *traA*. Interestingly, all branches displaying evidence of positive selection occur in males.
(PDF)

S4 Fig Population genetics of *V*. *emeryi* in the native range in Japan (A) and in the invasive range in the USA (B). Lines representing F_st_ molecular distances between queen clones connect each site. In the native range, each site had distinct queen and male clones, and F_st_ values among all populations were higher than 0.5. By contrast, all sites in the invasive range shared the same pair of clones, and F_st_ values among all populations were zero. Males show similar patterns of genetic differentiation (S3 Table). These data suggest low gene flow between sites in the native range, and a bottleneck in the invasive range, both demographic scenarios conducive to inbreeding.
(PNG)

S1 Data Linkage map of *V*. *emeryi* and genotype data. Homozygous and heterozygous sites are coded as 'A' and 'H', with missing values as '-'. Individuals are coded as phenotype-family-individual_id. Markers associated with QTLs are highlighted in blue.
(XLSX)

S2 Data Microsatellite genotypes of all 181 queens and 183 males (adult males and spermathecas) in Japan (A-E) and USA (F-H). Samples with an asterisk showed different genotypes from dominant clones at gray highlighted loci.
(XLSX)

S1 Table Offspring produced by outbred queens. In contrast to inbred crosses (Table 1), no diploid male offspring were produced during the experimental period.
(DOCX)

S2 Table Microsatellite markers developed for *V*. *emeryi* collected in Japan and USA. The observed size range, number of alleles per locus (*Na*), and frequency of the most common allele (*f*) were estimated from 126 queens and 122 males from Japan, and from 55 queens and 61 males from the USA. The number of alleles was different for Japan and USA. Observed and expected heterozygosities (*Ho* and *He*) were estimated with GENALex version 6.5 [61] using queens from Japan and USA. *Ta* is the annealing temperature for PCR.
(DOCX)

S3 Table Results of AMOVA (analysis of molecular variance) between populations in the native range. For diploid female samples, F_ST_ values were used to calculate genomic distance among populations, whereas Phi_PT_ values were used for haploid males (top of matrix, grey background). Both values were computed using pairwise genetic distances in GenAlEx version 6.5. Because GenAlex cannot use samples with mixed ploidy we separately calculated these values using (a) females in native area, (b) males in native area. Probabilities based on 999 permutations are shown above the diagonal. High F_ST_/Phi_PT_ values appeared in both females and males in the native area (female: 0.94 ± 0.05 SD, male: 0.98 ± 0.02 SD) suggesting rare gene flow among populations. In the invasive range, all queen clones were identical. Some spermathecal contents contained queen alleles, making it difficult to correctly genotype males, particularly in the invasive range where sample sizes were smaller. However, discounting the possibly of contaminated samples, the same male clone was present at high frequency in all three invasive populations (S4 Table).
(DOCX)

S4 Table Microsatellite genotypes of dominant queen and male clones in Japan (A-E) and the USA (F-H). Numbers within give the number of colonies collected at each site. In total, 181 queens and 183 males or spermathecal contents were genotyped. Numbers give lengths of PCR products at each microsatellite locus. All of the queens had the same genotypes at all microsatellite loci except site C (Japan), where seven of the 24 queens had genotypes that differed from that of the dominant queen clone (see S2 Data). In total, 35 adult males and 148 spermathecas were genotyped. Twenty-two spermathecas may have been contaminated with female tissue because some of the loci showed female specific alleles. In no case did we observe alleles in the spermathecal microsatellite profiles that were different from either the male or female clone, suggesting that the additional peaks were due to contamination, rather than to genuine polymorphism in the population (see S2 Data). However, we conservatively treated these 22 samples as different clonal lineages from dominant clone type, even though only one of the twelve loci showed a different genotype. Thus, the percentage of dominant male clones was likely significantly underestimated. Interestingly, queens were homozygous at all microsatellite loci, suggesting that parthenogenesis in this species results in large-scale loss of heterozygosity, except perhaps at sex determination loci.
(DOCX)

S1 Script R script for QTL analysis. Conducts a two-locus QTL analysis using R/QTL [51].
(TXT)
