# Supplementary figures and images for: QTL Mapping of Sex Determination Loci Supports an Ancient Pathway in Ants and Honey Bees
Source: PLoS Genet. 2015 Nov 6;11(11):e1005656. doi: 10.1371/journal.pgen.1005656 (PMC4636138; doi:10.1371/journal.pgen.1005656)

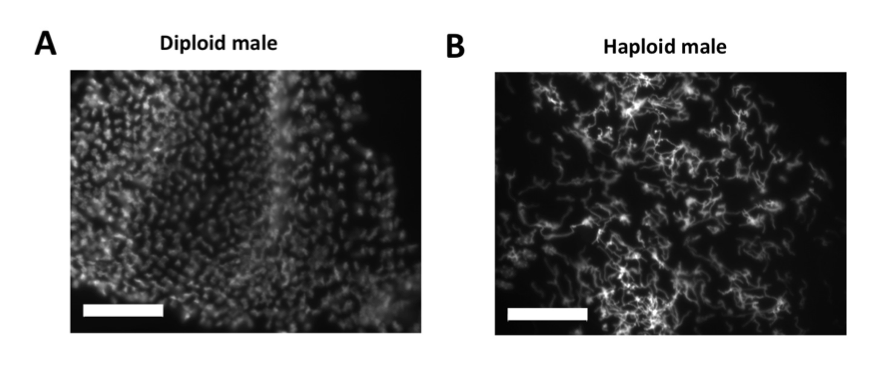

Supplement: S1 Fig — (A) males produced by sib-mated queens and (B) androgenetic males, stained with DAPI (32 and 16 males of each kind were dissected). Only nuclei of glands were observed in diploid males, whereas sperm (fibrous tissue) could be seen in androgenetic haploid males. Diploid males perform no work and do not produce sperm, suggesting that they are a major cost to the colony. Scale bar represents 50μm. (PNG) [file pgen.1005656.s001.png]

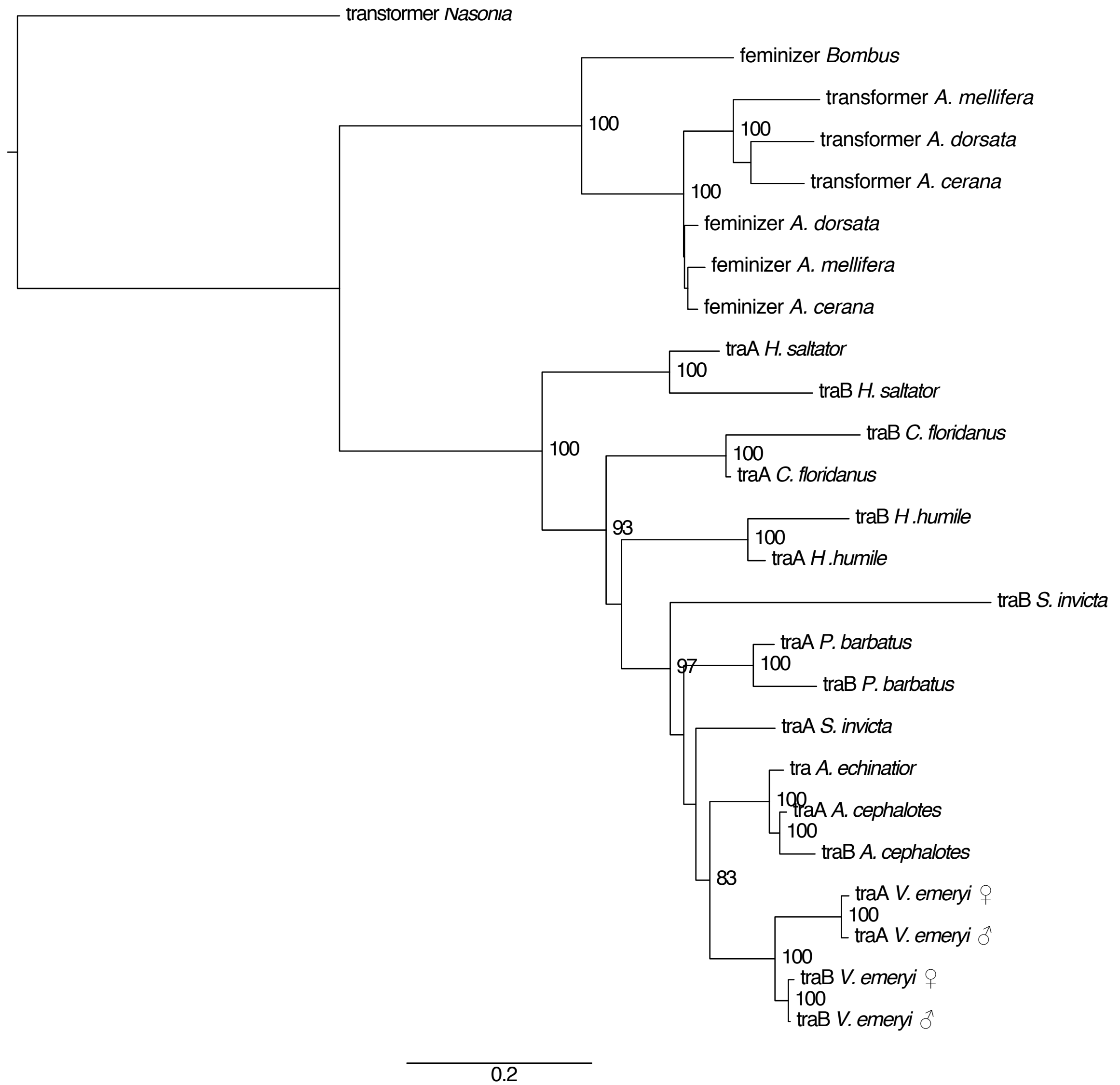

Supplement: S2 Fig — In the honey bee the two transformer homologs are called csd and fem. In other species these genes are referred to as tra homologs, because they are not functionally characterized. The tra homologs of V. emeryi are more similar to each other than to those of other species, consistent with frequent gene conversion [13]. With the exception of V. emeryi genes, all other sequences are as in Fig 1 in [13]. The alignment was made using codon sequences in MAFFT [55] and the tree was computed using RAXML [56] under the GTR+G model with 100 bootstrap replicates. The scale bar is in substitutions per site. (PDF) [file pgen.1005656.s002.pdf]

## Codon 169

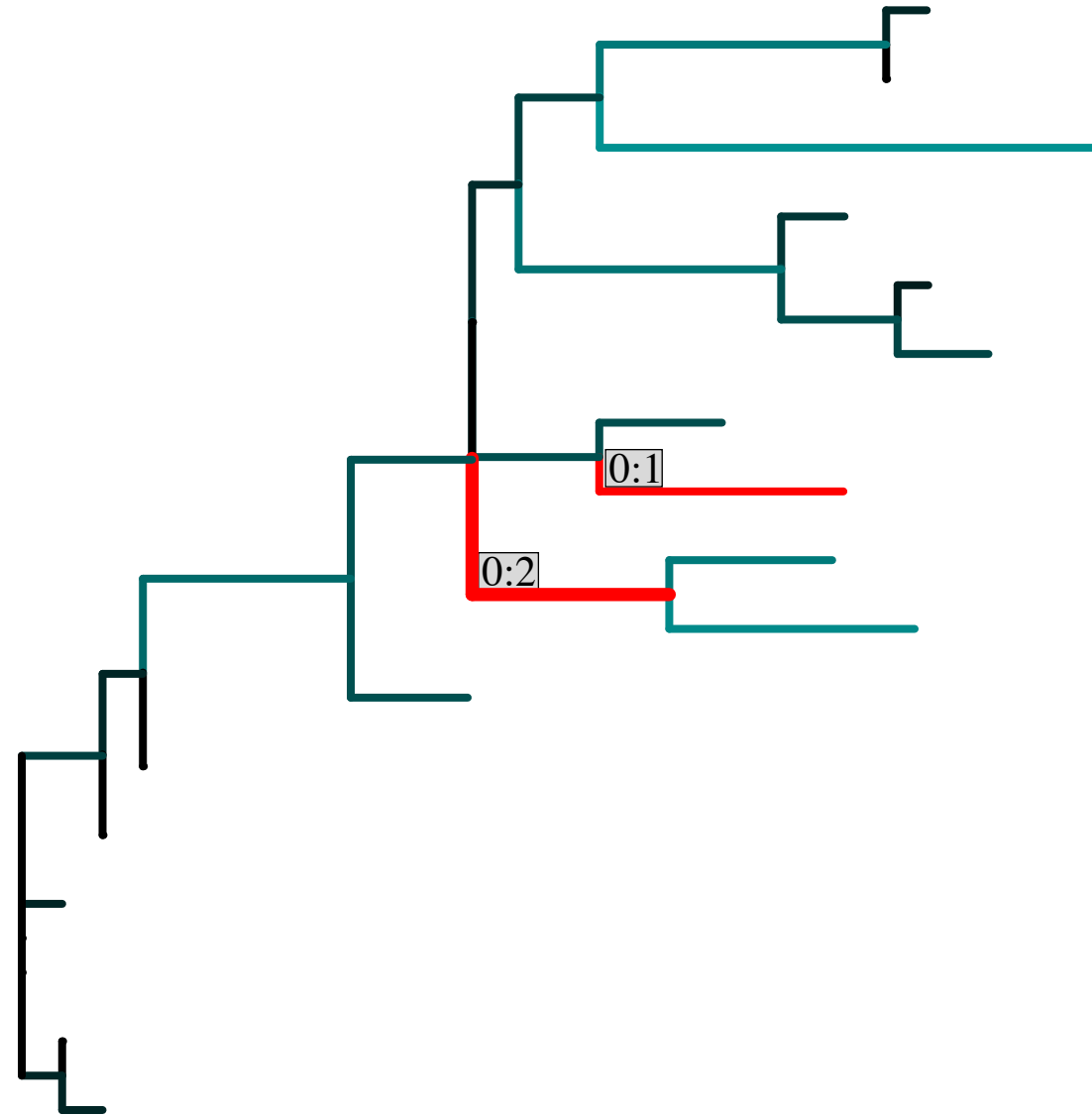

## Codon 207

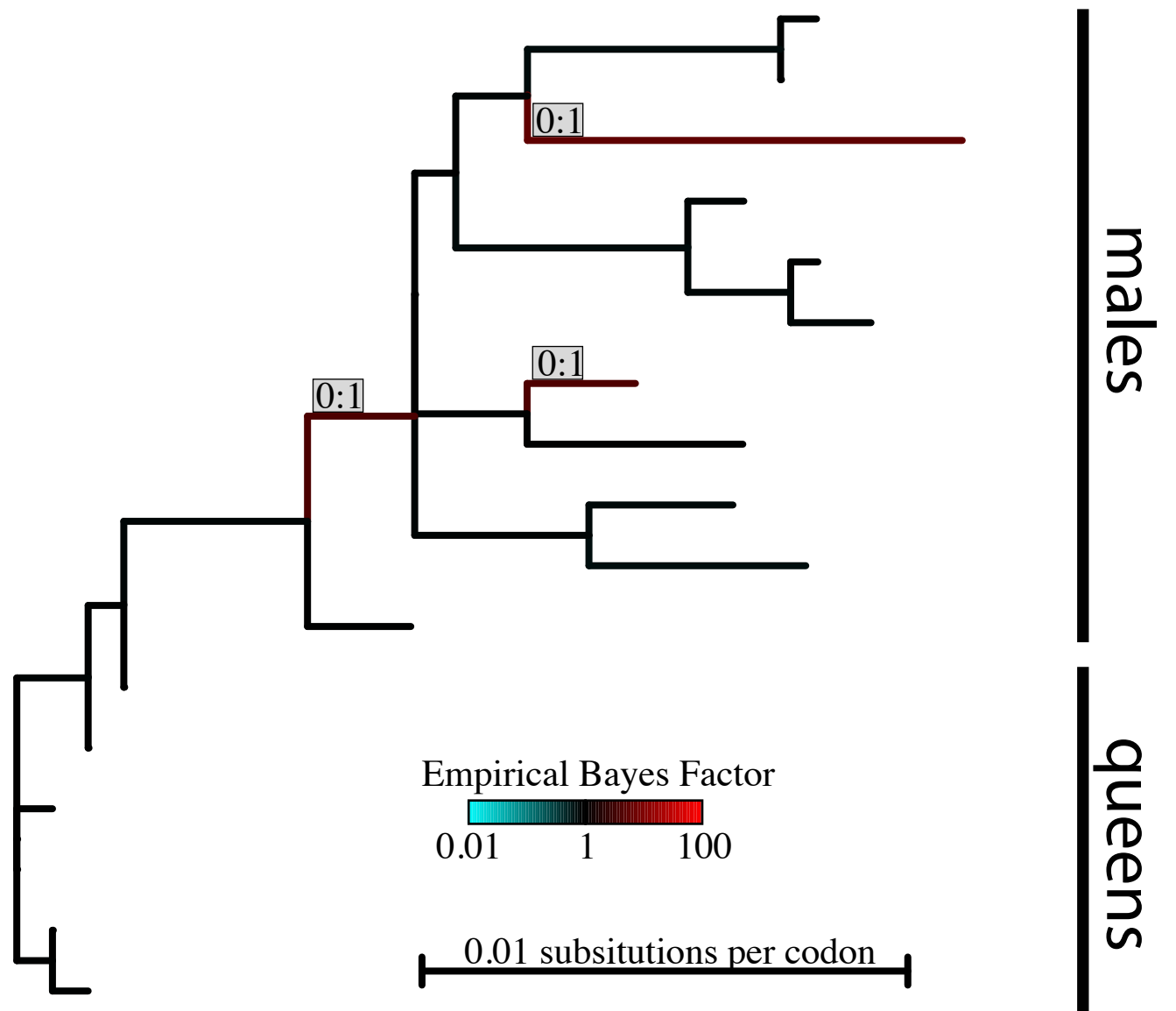

Supplement: S3 Fig — Interestingly, all branches displaying evidence of positive selection occur in males. (PDF) [file pgen.1005656.s003.pdf]

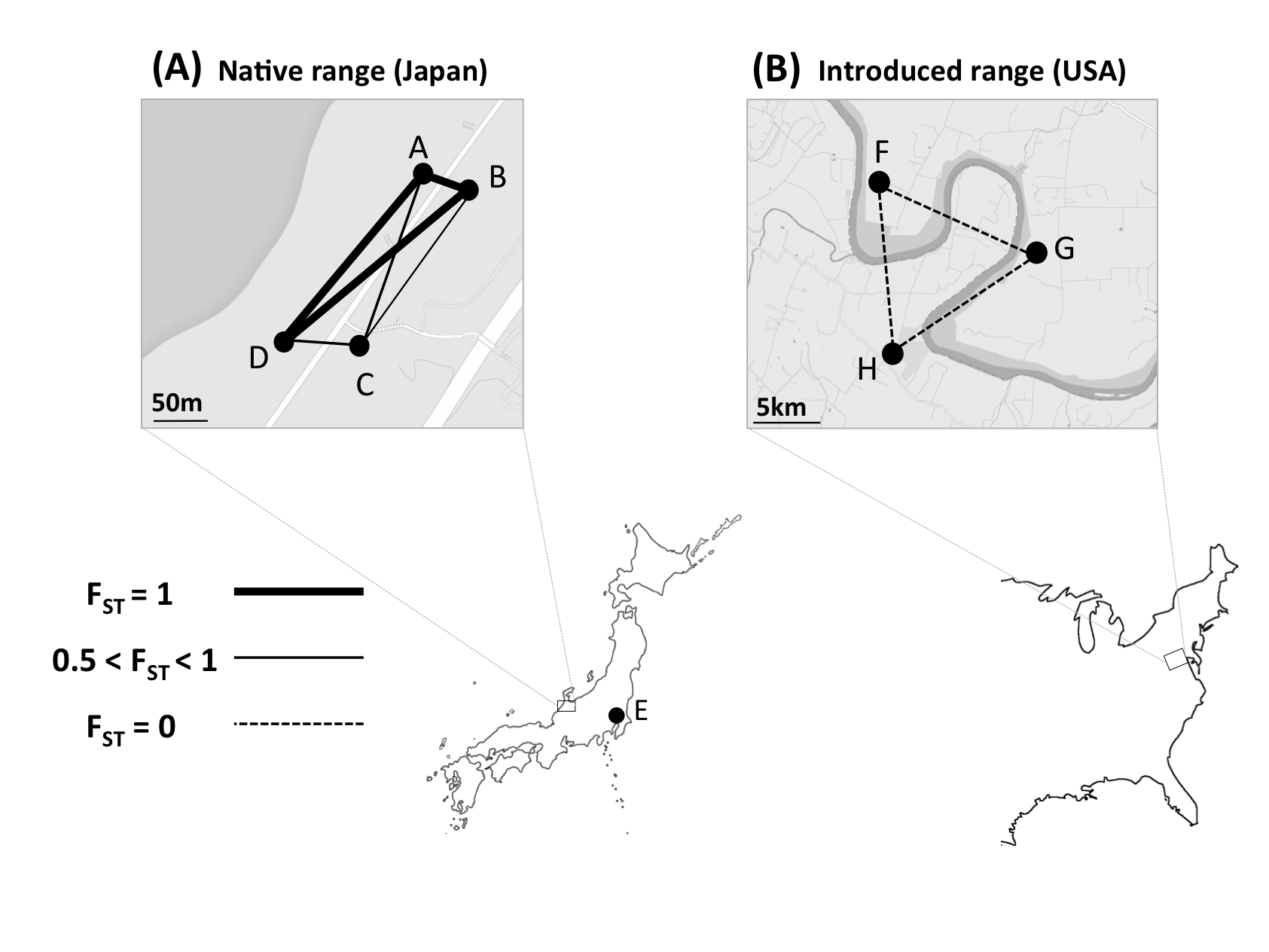

Supplement: S4 Fig — Lines representing Fst molecular distances between queen clones connect each site. In the native range, each site had distinct queen and male clones, and Fst values among all populations were higher than 0.5. By contrast, all sites in the invasive range shared the same pair of clones, and Fst values among all populations were zero. Males show similar patterns of genetic differentiation (S3 Table). These data suggest low gene flow between sites in the native range, and a bottleneck in the invasive range, both demographic scenarios conducive to inbreeding. (PNG) [file pgen.1005656.s004.png]
