# Supplementary material for: QTL Mapping of Sex Determination Loci Supports an Ancient Pathway in Ants and Honey Bees
Source: PLoS Genet. 2015 Nov 6;11(11):e1005656. doi: 10.1371/journal.pgen.1005656 (PMC4636138; doi:10.1371/journal.pgen.1005656)
Supplement: S1 Table — In contrast to inbred crosses (Table 1), no diploid male offspring were produced during the experimental period. (DOCX) [file pgen.1005656.s007.docx]

**S1 Table. Offspring produced by outbred queens.** In contrast to inbred crosses (Table 1), no diploid male offspring were produced during experimental period.

|  |  |  |  |  |
| --- | --- | --- | --- | --- |
|  | Number of offspring | |  |  |
| Queen ID | Female | Worker | Diploid male | Total |
| 1 | 4 | 64 | 0 | 68 |
| 2 | 0 | 95 | 0 | 95 |
| 3 | 0 | 244 | 0 | 244 |
| 4 | 0 | 168 | 0 | 168 |
| 5 | 0 | 151 | 0 | 151 |
| 6 | 0 | 108 | 0 | 108 |
| 7 | 0 | 130 | 0 | 130 |
| 8 | 0 | 55 | 0 | 55 |
| 9 | 0 | 62 | 0 | 62 |
| 10 | 0 | 101 | 0 | 101 |
| 11 | 0 | 89 | 0 | 89 |
| 12 | 0 | 62 | 0 | 62 |
| 13 | 0 | 77 | 0 | 77 |
| 14 | 0 | 48 | 0 | 48 |
| 15 | 0 | 87 | 0 | 87 |
| 16 | 0 | 110 | 0 | 110 |
| 17 | 0 | 87 | 0 | 87 |
|  |  |  |  |  |
