# Supplementary material for: QTL Mapping of Sex Determination Loci Supports an Ancient Pathway in Ants and Honey Bees
Source: PLoS Genet. 2015 Nov 6;11(11):e1005656. doi: 10.1371/journal.pgen.1005656 (PMC4636138; doi:10.1371/journal.pgen.1005656)
Supplement: S2 Table — The observed size range, number of alleles per locus (Na), and frequency of the most common allele (f) were estimated from 126 queens and 122 males from Japan, and from 55 queens and 61 males from the USA. The number of alleles was different for Japan and USA. Observed and expected heterozygosities (Ho and He) were estimated with GENALex version 6.5 [61] using queens from Japan and USA. Ta is the annealing temperature for PCR. (DOCX) [file pgen.1005656.s008.docx]

**S2 Table. | Microsatellite developed for *V. emeryi* collected in Japan and USA**. The observed size range, number of alleles per locus (*Na*), frequency of the most common allele (*f*) were estimated from 126 queens and 122 males from Japan, 55 queens and 61 males from USA. The number of alleles was different for Japan and USA. Observed and expected heterozygosities (*Ho* and *He*) were estimated by GENALex version 6.5 using queens from Japan and USA. *Ta* is the annealing temperature for PCR.

| Locus | Size | *Na* |  |  | *f* |  |  |  |  |  |  | *Ho* |  |  | *He* |  |  | *Ta* |  |
| --- | --- | --- | --- | --- | --- | --- | --- | --- | --- | --- | --- | --- | --- | --- | --- | --- | --- | --- | --- |
|  |  | JPN | USA |  | JPN | | | USA | | |  | JPN | USA |  | JPN | USA |  | (℃) | Primers (5'-3') |
|  |  |  |  |  | ♀ / ♂ | | | ♀ / ♂ | | |  |  |  |  |  |  |  |  |  |
| CT_00394 | 226-236 | 5 | 2 |  | 0.637 | / | 0.755 | 1.000 | / | 0.967 |  | 0.255 | 0.000 |  | 0.522 | 0.000 |  | 58 | F: GGTGGCCTCCCATTATTTGT R: CCTAGCATCGGGTGCCTTAT |
| CT_01527 | 200-210 | 4 | 1 |  | 0.810 | / | 0.426 | 1.000 | / | 1.000 |  | 0.000 | 0.000 |  | 0.308 | 0.000 |  | 58 | F: ATTTGGCGGTAGGACTTTCC R: ATTTCGTCCCGACGTACTTG |
| CT_02726 | 166-178 | 5 | 2 |  | 0.571 | / | 0.393 | 1.000 | / | 0.951 |  | 0.000 | 0.000 |  | 0.582 | 0.000 |  | 58 | F: CGGTAGGGAGGAGAtGGTTT R: AGATTTCGCGGCCTATCTCT |
| CT_03471 | 185-220 | 7 | 2 |  | 0.575 | / | 0.410 | 1.000 | / | 0.984 |  | 0.008 | 0.000 |  | 0.583 | 0.000 |  | 58 | F: CATTTGATCACCGCCTCTTT R: GCCGCGTGTTAATTGTCATA |
| CT_04024 | 235-272 | 7 | 2 |  | 0.357 | / | 0.531 | 1.000 | / | 0.984 |  | 0.000 | 0.000 |  | 0.733 | 0.000 |  | 58 | F: AAGAACAATATTTCAATTAACCGATTT R: AGGACCAGATGTTCCTGGTG |
| CT_06089 | 201-205 | 3 | 2 |  | 0.754 | / | 0.590 | 1.000 | / | 0.967 |  | 0.000 | 0.000 |  | 0.392 | 0.000 |  | 58 | F: AAAACGCAGAGTAGGCCAAA R: GCGACACTTGCATTTTACGA |
| CT_07999 | 242-251 | 0 | 2 |  | - | / | 0.000 | 1.000 | / | 1.000 |  | - | 0.000 |  | - | 0.000 |  | 58 | F: ATAATCGTGGCGTCAAGCA R: CGTTTAGTTTCAATCACATGACG |
| CT_09869 | 164-172 | 5 | 2 |  | 1.000 | / | 0.286 | - | / | 0.984 |  | 0.000 | - |  | 0.000 | - |  | 58 | F: CGCAGTATGGAAGGAACTGG R: ACTTTTGTTGGGGTCTCCTG |
| CT_13446 | 252-168 | 3 | 1 |  | 1.000 | / | 0.686 | 0.000 | / | 1.000 |  | 0.000 | 0.000 |  | 0.000 | 0.000 |  | 58 | F: AGTGCGCAAGGTGTTGTGT R: TTCTCAGGGAAGCGATTTTC |
| CT_28597 | 250-263 | 5 | 2 |  | 0.519 | / | 0.776 | 1.000 | / | 0.967 |  | 0.000 | 0.000 |  | 0.499 | 0.000 |  | 58 | F: AGGCGGTAGGCAGCTACTTT R: TCTCTCtCTTTTCGTGATGCAA |
| CT_21332 | 168-179 | 4 | 2 |  | 1.000 | / | 0.365 | 1.000 | / | 1.000 |  | 0.000 | 0.000 |  | 0.000 | 0.000 |  | 58 | F: ACGGGCAAATAACACTGACC R: TATTCGCTGTCGCCAATAAA |
| L-5 | 109-113 | 3 | 2 |  | 0.810 | / | 0.787 | 1.000 | / | 0.984 |  | 0.000 | 0.000 |  | 0.308 | 0.000 |  | 55 | F: ATTAAGTCAATTTTCAATGCCGCAA R: CTGATGATGCATCTGTGGGATCT |
| Vems-78 | 108-118 | 2 | 2 |  | 1.000 | / | 1.000 | 1.000 | / | 0.984 |  | 0.000 | 0.000 |  | 0.000 | 0.000 |  | 55 | F: CCCACGGTAACCCTCGAGAAC R: AGCGGAGAAACAGGCGAGAAA |
