# Supplementary material for: QTL Mapping of Sex Determination Loci Supports an Ancient Pathway in Ants and Honey Bees
Source: PLoS Genet. 2015 Nov 6;11(11):e1005656. doi: 10.1371/journal.pgen.1005656 (PMC4636138; doi:10.1371/journal.pgen.1005656)
Supplement: S3 Table — For diploid female samples, FST values were used to calculate genomic distance among populations, whereas PhiPT values were used for haploid males (top of matrix, grey background). Both values were computed using pairwise genetic distances in GenAlEx version 6.5. Because GenAlex cannot use samples with mixed ploidy we separately calculated these values using (a) females in native area, (b) males in native area. Probabilities based on 999 permutations are shown above the diagonal. High FST/PhiPT values appeared in both females and males in the native area (female: 0.94 ± 0.05 SD, male: 0.98 ± 0.02 SD) suggesting rare gene flow among populations. In the invasive range, all queen clones were identical. Some spermathecal contents contained queen alleles, making it difficult to correctly genotype males, particularly in the invasive range where sample sizes were smaller. However, discounting the possibly of contaminated samples, the same male clone was present at high frequency in all three invasive populations (S4 Table). (DOCX) [file pgen.1005656.s009.docx]

**S3 Table. | Result of AMOVA (Analysis of molecular variance) between populations in native range.**

| (A) Females in native area | |  |  |  |  |
| --- | --- | --- | --- | --- | --- |
| A | B | C | E | D |  |
| 0.000 | 0.001 | 0.001 | 0.001 | 0.001 | A |
| 1.000 | 0.000 | 0.001 | 0.001 | 0.001 | B |
| 0.913 | 0.892 | 0.000 | 0.001 | 0.001 | C |
| 0.934 | 0.936 | 0.849 | 0.000 | 0.001 | E |
| 1.000 | 1.000 | 0.928 | 0.955 | 0.000 | D |
|  |  |  |  |  |  |
|  |  |  |  |  |  |
| (B) Males in native area | |  |  |  |  |
| A | B | C | E | D |  |
| 0.000 | 0.001 | 0.001 | 0.001 | 0.001 | A |
| 1.000 | 0.000 | 0.001 | 0.001 | 0.001 | B |
| 0.913 | 0.892 | 0.000 | 0.001 | 0.001 | C |
| 0.934 | 0.936 | 0.849 | 0.000 | 0.001 | E |
| 1.000 | 1.000 | 0.928 | 0.955 | 0.000 | D |
