# Supplementary material for: QTL Mapping of Sex Determination Loci Supports an Ancient Pathway in Ants and Honey Bees
Source: PLoS Genet. 2015 Nov 6;11(11):e1005656. doi: 10.1371/journal.pgen.1005656 (PMC4636138; doi:10.1371/journal.pgen.1005656)
Supplement: S4 Table — Numbers within give the number of colonies collected at each site. In total, 181 queens and 183 males or spermathecal contents were genotyped. Numbers give lengths of PCR products at each microsatellite locus. All of the queens had the same genotypes at all microsatellite loci except site C (Japan), where seven of the 24 queens had genotypes that differed from that of the dominant queen clone (see S2 Data). In total, 35 adult males and 148 spermathecas were genotyped. Twenty-two spermathecas may have been contaminated with female tissue because some of the loci showed female specific alleles. In no case did we observe alleles in the spermathecal microsatellite profiles that were different from either the male or female clone, suggesting that the additional peaks were due to contamination, rather than to genuine polymorphism in the population (see S2 Data). However, we conservatively treated these 22 samples as different clonal lineages from dominant clone type, even though only one of the twelve loci showed a different genotype. Thus, the percentage of dominant male clones was likely significantly underestimated. Interestingly, queens were homozygous at all microsatellite loci, suggesting that parthenogenesis in this species results in large-scale loss of heterozygosity, except perhaps at sex determination loci. (DOCX) [file pgen.1005656.s010.docx]

**S4 Table. | Microsatellite genotype of dominant queen and male clones in Japan (A-E) and USA (F-H).**

| USA | F (6) | |  |  | G (8) | |  |  | H (3) | |  |
| --- | --- | --- | --- | --- | --- | --- | --- | --- | --- | --- | --- |
| Locus | Queen | | Male |  | Queen | | Male |  | Queen | | Male |
| L-5 | 111 | 111 | 113 |  | 111 | 111 | 113 |  | 111 | 111 | 113 |
| Vems 78 | 108 | 108 | 118 |  | 108 | 108 | 118 |  | 108 | 108 | 118 |
| CT_03471 | 196 | 196 | 220 |  | 196 | 196 | 220 |  | 196 | 196 | 220 |
| CT_09869 | 164 | 164 | 172 |  | 164 | 164 | 172 |  | 164 | 164 | 172 |
| CT_28597 | 253 | 253 | 263 |  | 253 | 253 | 263 |  | 253 | 253 | 263 |
| CT_13446 | null | null | 168 |  | null | null | 168 |  | null | null | 168 |
| CT_07999* | 243 | 243 | 251 |  | 243 | 243 | 251 |  | 243 | 243 | 251 |
| CT_04024 | 251 | 251 | 254 |  | 251 | 251 | 254 |  | 251 | 251 | 254 |
| CT_02726 | 178 | 178 | 174 |  | 178 | 178 | 174 |  | 178 | 178 | 174 |
| CT_00934 | 236 | 236 | 226 |  | 236 | 236 | 226 |  | 236 | 236 | 226 |
| CT_21332 | 168 | 168 | 179 |  | 168 | 168 | 179 |  | 168 | 168 | 179 |
| CT_06089 | 205 | 205 | 203 |  | 205 | 205 | 203 |  | 205 | 205 | 203 |
| Total individuals | 23 |  | 26 |  | 10 |  | 7 |  | 22 |  | 28 |
| Dominant clones | 100% | | 73.1% |  | 100.0% | | 57.0% |  | 100.0% | | 96.4% |

| Japan | A (10) | |  |  | B (11) | | |  | |  | | C (8) | | | | |  | |  | | D (10) | | | |  |  | | E (11) | | | |  |
| --- | --- | --- | --- | --- | --- | --- | --- | --- | --- | --- | --- | --- | --- | --- | --- | --- | --- | --- | --- | --- | --- | --- | --- | --- | --- | --- | --- | --- | --- | --- | --- | --- |
| Locus | Queen | | Male |  | Queen | | | Male | |  | | Queen | | | | | Male | |  | | Queen | | | Male | |  | Queen | | | | Male | |
| L-5 | 111 | 111 | 113 |  | 111 | 111 | | 113 |  | | 111 | | 111 | | | 113 | |  | | | 109 | | 109 | 111 | |  | 111 | | | 111 | 113 | |
| Vems 78 | 108 | 108 | 118 |  | 108 | 108 | | 118 |  | | 108 | | 108 | | | 118 | |  | | | 108 | | 108 | 118 | |  | 108 | | | 108 | 118 | |
| CT_03471 | 196 | 196 | 200 |  | 187 | 187 | | 210 |  | | 185 | | 185 | | | 190 | |  | | | 185 | | 185 | 190 | |  | 185 | | | 185 | 200 | |
| CT_09869 | 164 | 164 | 172 |  | 164 | 164 | | 183 |  | | 164 | | 164 | | | 167 | |  | | | 164 | | 164 | null | |  | 164 | | | 164 | 168 | |
| CT_28597 | 250 | 250 | 263 |  | null | null | | 263 |  | | null | | null | | | 263 | |  | | | null | | null | null | |  | 252 | | | 252 | 260 | |
| CT_13446 | null | null | Null |  | null | null | | 162 |  | | null | | null | | | 162 | |  | | | null | | null | null | |  | null | | | null | 168 | |
| CT_01527* | 210 | 210 | 215 |  | 210 | 210 | | 215 |  | | 200 | | 200 | | | 205 | |  | | | 210 | | 210 | 210 | |  | 210 | | | 210 | 210 | |
| CT_04024 | 235 | 235 | 243 |  | 250 | 250 | | 252 |  | | 235 | | 235 | | | 243 | |  | | | 272 | | 272 | null | |  | 260 | | | 260 | 250 | |
| CT_02726 | 176 | 176 | 184 |  | 166 | 166 | | 172 |  | | 166 | | 166 | | | 176 | |  | | | 166 | | 166 | 176 | |  | 175 | | | 175 | 172 | |
| CT_00934 | 230 | 230 | 232 |  | null | null | | null |  | | 230 | | 230 | | | 232 | |  | | | 235 | | 235 | 233 | |  | 230 | | | 236 | 232 | |
| CT_21332 | 168 | 168 | 179 |  | 168 | 168 | | null |  | | 168 | | 168 | | | 179 | |  | | | 168 | | 168 | null | |  | 168 | | | 168 | 174 | |
| CT_06089 | 203 | 203 | 201 |  | 203 | 203 | | 203 |  | | 203 | | 203 | | | 201 | |  | | | 202 | | 202 | 202 | |  | 203 | | | 203 | 201 | |
| Total individuals | 28 |  | 28 |  | 24 | |  | 24 | |  | | 24 | |  | | | 24 | |  | | 24 | |  | 24 | |  | 26 | | |  | 22 | |
| Dominant clones | 100% | | 92.9% |  | 100.0% | | | 79.2% | |  | | 70.8% | | | 100.0% | | | | |  | | 100.0% | | 100.0% | | |  | | 100.0% | | 81.8% | |
